# Endovascular Treatment of Acute Lower Limb Ischemia Associated with Cocaine Use: A Scoping Review

**DOI:** 10.7759/cureus.54672

**Published:** 2024-02-22

**Authors:** Julián Andrés Muñoz Durán, Santiago Echeverri Isaza, José Migue Hidalgo Oviedo, Emilio Sanin, Sergio Alvarez-Vallejo, Vanessa García Gómez

**Affiliations:** 1 Department of Interventional Radiology, Universidad de Antioquia, Medellin, COL; 2 Department of Interventional Radiology, Hospital Pablo Tobón Uribe, Medellin, COL; 3 Department of Radiology, Hospital Pablo Tobón Uribe, Medellin, COL

**Keywords:** crack, lower limb, ischemia, acute, cocaine

## Abstract

The endovascular treatment of acute lower limb ischemia associated with cocaine use is an underexplored topic. This scoping review aims to map existing information and point out potential knowledge gaps for future research. We searched databases with a strategy of terms and keywords (Cocaine, Acute, Lower Limb, and Ischemia) for articles related to acute ischemia in the lower limbs and cocaine use. We established eligibility and exclusion criteria and searched without restrictions on language or date of publication. We obtained five case reports published between 2004 and 2015. Most of the patients were men with an average age of 38 years. Treatments were heterogeneous. Most patients showed improvement after surgical thrombectomy. The sample size and variety of interventions limit the generalizability of the results, so it is necessary to do more studies with robust methodologies to standardize treatments and improve the understanding of the condition.

## Introduction and background

‘Khoka’ is an Aymara word meaning ‘The Tree.’ Coca (*Erythroxylum coca* Lam and *Erythroxylum novogranatense* (Morris)) is an indigenous South American plant, whose leaves have long been integral to Andean culture for their ritual, social, and physiological uses. This plant contains several compounds, the most notable being cocaine, a psychoactive component first isolated in 1860 by Dr. Albert Niemann from cultivated coca leaves [1].

Cocaine is known to induce vasoconstriction and thrombosis in arteries that supply vital organs such as the myocardium, brain, muscles, kidneys, uterus, and intestines [2-4]. Its vasoactive properties stem from the accumulation of noradrenaline, disruption of prostaglandin production leading to vasospasm, and the activation of platelets resulting in thrombus formation [5,6]. The vascular damage may be exacerbated by impurities and adulterants, like arsenic, associated with thromboangiitis obliterans [7]. Acute lower limb ischemia linked to recent cocaine use is believed to primarily involve vasoconstriction in the vascular bed of skeletal muscles, leading to tissue ischemia, rhabdomyolysis, and thrombosis [8,9].

Over the past century, cocaine, a highly addictive substance used illicitly for its psychoactive effects, has emerged as a significant public health issue [10]. Its adverse effects on the cardiovascular system, including ischemia and arrhythmias, are well-documented [11]. Less common conditions, such as thrombosis or thromboembolism in the pulmonary vasculature [12,13] and lower limb ischemia, though less frequently reported, are also of concern.

Therefore, the extent and nature of information available in the literature on endovascular treatments for acute lower limb ischemia associated with cocaine use, the outcomes of various interventions, and the main demographic characteristics of affected patients remain unclear. To address these uncertainties, a scoping review was conducted to systematically map the available literature, with a particular focus on endovascular treatment, and to identify potential gaps in current knowledge to guide future research.

## Review

Materials and methods

Design

A scoping review of the peer-reviewed literature was conducted to map the existing literature and identify knowledge gaps, with a particular emphasis on the endovascular treatment of acute lower limb ischemia associated with cocaine use. A scoping review was chosen for its suitability in synthesizing research on topics that are not extensively documented and where studies may vary significantly in methods or approaches. Additionally, the quality of these studies is not the primary focus, in line with the guidelines described by Arksey and O'Malley [14] and refined by Levac et al. [15].

This scoping review was carried out in six stages, detailed as follows: identifying the research question, identifying relevant studies, study selection, charting the data, collating, summarizing, and reporting results and consultation. Additionally, the Preferred Reporting Items for Systematic Reviews and Meta-Analyses extension for Scoping Reviews (PRISMA-ScR) guidelines [16] were utilized to inform the reporting of results, promoting standardization and rigor in our approach.

Research Question

We formulated the following primary research question to guide our examination of the literature: What is the extent, scope, and nature of the evidence available in the literature regarding the endovascular treatment of acute lower limb ischemia associated with cocaine use? To comprehensively address this central theme, we also explored the following sub-questions: What pharmacological treatment strategies are being used in these cases?, What are the demographic characteristics of patients suffering from this condition, and is there a correlation between these characteristics and treatment outcomes?, and What are the outcomes for patients following treatment?. These sub-questions were designed to facilitate a structured and thorough review of the literature, enabling us to synthesize data and clearly delineate established areas of knowledge.

Search Strategy

A peer-reviewed search of indexed articles was conducted by at least two researchers, utilizing key terms combined with Boolean operators in databases PubMed, Scopus, LILACS (Latin American and Caribbean Health Sciences Literature), and Embase (Excerpta Medica Database). This approach follows the recommendations by Levac et al. [15] for an iterative strategy, allowing for the refinement of the search as needed. We focused on keyword groups related to the topic: Cocaine, Acute, Lower Limb, and Ischemia.

The specific search terms for PubMed were: ('cocaine'[MeSH Terms] OR 'cocaine'[All Fields] OR 'cocaine s'[All Fields] OR 'cocaines'[All Fields] OR 'cocainics'[All Fields]) AND ('acute'[All Fields] OR 'acutely'[All Fields] OR 'acutes'[All Fields]) AND ('lower extremity'[MeSH Terms] OR ('lower'[All Fields] AND 'extremity'[All Fields]) OR 'lower extremity'[All Fields] OR ('lower'[All Fields] AND 'limb'[All Fields]) OR 'lower limb'[All Fields]) AND ('ischaemia'[All Fields] OR 'ischemia'[MeSH Terms] OR 'ischemia'[All Fields] OR 'ischaemias'[All Fields] OR 'ischemias'[All Fields]), yielding 12 records. For Scopus: TITLE-ABS-KEY (cocaine AND acute AND lower AND limb AND ischemia), obtaining 13 records. In LILACS: cocaine AND acute AND lower limb AND ischemia AND (db:'LILACS') with one record. In EMBASE: ('cocaine'/exp OR cocaine) AND acute AND ('lower limb'/exp OR 'lower limb' OR (lower AND ('limb'/exp OR limb)) AND ('ischemia'/exp OR ischemia), resulting in 59 search results.

**Figure 1:**
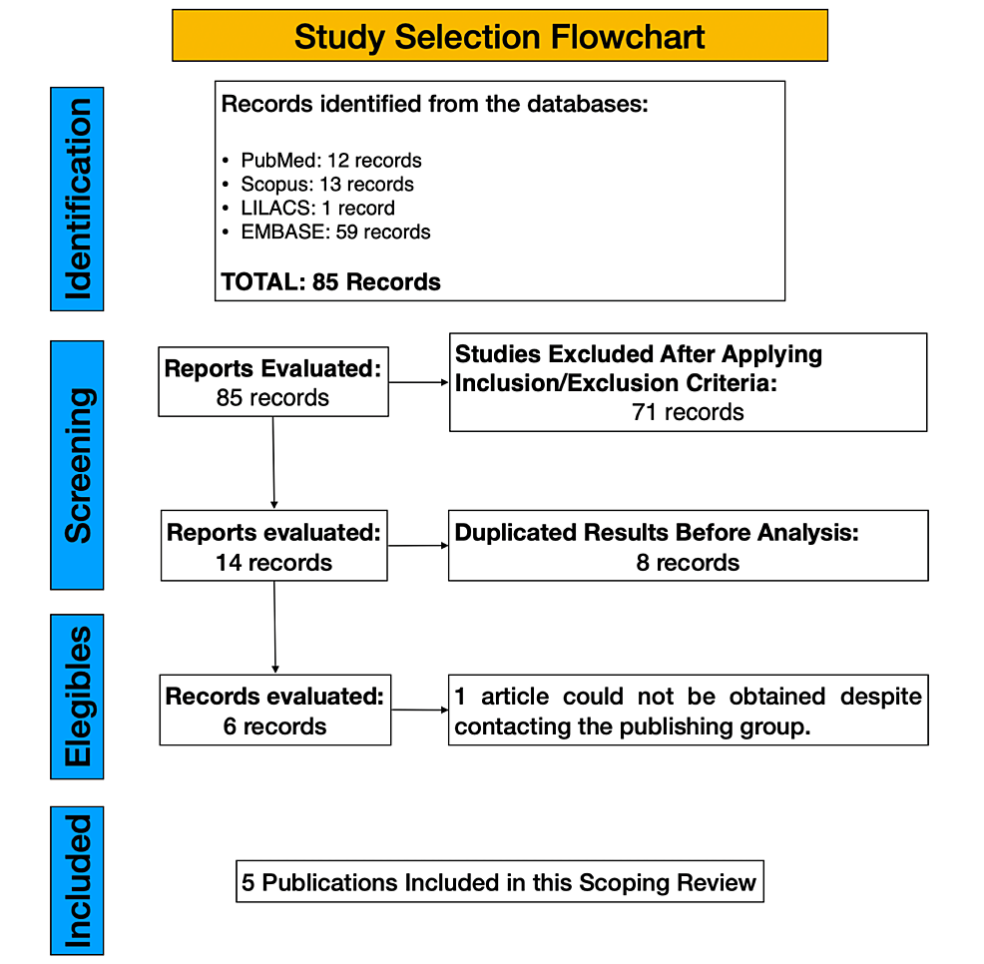
Study selection flowchart.

The initial search was conducted on August 20, 2023, without restrictions on language or publication date, to encompass the literature up to the current date. A second search was scheduled three months after the initial date to capture any newly published literature (Figure 1). We utilized Zotero reference management software (Corporation for Digital Scholarship, Vienna, Virginia, United States) to collect and organize bibliographic citations and to eliminate duplicate entries. Articles were selected based on predefined inclusion and exclusion criteria. Those not available in full text, even after attempting to contact the journal, were excluded, as were non-indexed articles and grey literature.

Study Selection

The review process was conducted in two stages to ensure a rigorous and reproducible selection of studies. Phase 1 was the Preliminary Review. In this phase, two review authors independently assessed titles and abstracts using predefined inclusion and exclusion criteria to ensure objectivity and reproducibility. Studies that did not meet these criteria were excluded at this stage. Phase 2 was the Full-Text Evaluation. This stage involved a detailed review of the full texts to confirm eligibility. To further reduce the risk of overlooking eligible studies, a third review author re-examined the studies excluded during the full-text review phase. In our study, there were no discrepancies or uncertainties in the selection process.

Inclusion criteria: Studies focusing on acute lower limb ischemia in the context of cocaine use, articles describing treatment modalities for acute lower limb ischemia associated with cocaine use, and publications in any language and without time restrictions. Conference posters published in scientific journals were included.

Exclusion criteria: Studies where the ischemic event is not directly related to acute cocaine use, as this is the central condition under study, articles that remained inaccessible even after attempting to contact the publisher, precluding analysis, and potentially eligible publications that do not provide sufficient information for inclusion in the review due to inadequate data or findings classification.

We believe that this methodology minimizes the risk of selection bias and ensures the retrieval of relevant articles in alignment with the objectives of this review.

Data Extraction

Data from the included articles were systematically charted following the guidelines outlined by Peter et al. [17]. Consistent with the nature of scoping reviews, the quality of the included studies was not formally assessed [18]. However, certain characteristics of the studies are described that may provide insights into the robustness of the available evidence. The data was synthesized and presented in tables, facilitating a compact overview of the research landscape and identifying trends in areas where the evidence is either strong or lacking.

The data were organized according to key variables pertinent to our research questions (Table 1). This included the author(s), year of publication, country of origin, type of study (e.g., clinical trial, cohort study, case-control, case report) and its main findings, patient characteristics including information such as age, gender distribution, and any other reported demographic characteristics, clinical details including clinical presentation, relevant medical history, and duration of symptoms, when available, treatment including specific procedures and pharmacological strategies which were categorized to enhance consistency (heparin use, intra-arterial therapy, systemic vasodilators, surgical thrombectomy, and nerve block), and follow-up including post-treatment outcomes, classified into categories such as necrosis, amputation, and improvement.

**Table 1:** Main characteristics of included studies

Author	Age/Gender of the patients	Clinical Presentation	Diagnostic Findings	Treatment	Outcome	Country of Origin/Language of the articles	Year of publication	Type
Collins et al. [5].	31 years old, male	Progressive claudication of 8 weeks progressing to severe ischemia of the 3rd toe of the left foot	Left tibiofibular occlusion without thrombus	Heparin with systemic vasodilator (Iloprost)	Improvement	Ireland/English	2008	Case Report
Zhou et al. [11].	42 years old, male	Acute left lower limb ischemia	Left common iliac artery thrombosis	Heparin with surgical thrombectomy	Improvement	United States/English	2004	Case Report
	49 years old, male	Acute right lower limb ischemia	Right external iliac artery thrombosis	Heparin with surgical thrombectomy	Improvement		2004	Case Report
	38 years old, female	Acute right lower limb ischemia	Right superficial femoral artery thrombosis	Heparin with surgical thrombectomy	Improvement		2004	Case Report
	46 years old, male	Acute left lower limb ischemia	Left popliteal artery thrombosis	Heparin with thrombolysis (Urokinase)	Improvement		2004	Case Report
McMullin et al. [19].	34 years old, female	Pain and acute ischemia in the 4 limbs of 4 days of evolution	Severe vasospasm in 4 extremities	Heparin with systemic vasodilators (Nitroglycerin and Iloprost), Intraarterial therapy (Iloprost, nitroglycerin y guanethidine), Balloon angioplasty, Surgical thrombectomy.	Necrosis (treated conservatively).	United Kingdom/English	2015	Case Report
Debien et al. [20].	31 years old, male	Stroke and acute ischemia of the left foot	Acute ischemia of the first and fifth fingers, left lower limb	Sciatic Nerve Block and Intra-Arterial Therapy (Ropivacaine)	Amputation	France/French	2005	Case Report
Seinturier et al. [21].	35 years old, female	Claudication for one month and then acute ischemia of the left leg.	Left Popliteal Arterial Thrombosis and Probable Buerger's Disease	Systemic heparin	Improvement	France/French	2006	Case Report

Results

This review encompassed five case reports published between 2004 and 2015, each describing incidents of acute arterial ischemia associated with cocaine use. The geographical distribution of these articles was as follows: United States (one article, 20%), United Kingdom (one article, 20%), Ireland (one article, 20%), and France (two articles, 40%). The predominant language across these publications is English, accounting for three of the articles. Unfortunately, one additional article could not be included in this review due to inaccessibility, despite our efforts to obtain it from the respective journal.

The majority of the patients in these case reports were male, accounting for five out of eight patients (62%), with an average age of 38.25 years, ranging from 31 to 49 years. This trend might indicate a higher susceptibility among men in their third and fourth decades of life to this complication. However, it is important to note that the small sample size of these case reports limits the robustness of any conclusions that can be drawn from this observation.

The available literature on the endovascular treatment of acute lower limb ischemia associated with cocaine use is notably limited, primarily due to the small number of patients and the absence of methodologically robust studies. Currently, only case reports contribute to this research area. These reports often present heterogeneous treatment strategies, with variability in drug administration routes and imaging findings. Standardized reporting in these aspects is either lacking or insufficiently detailed. The undeniable lack of uniformity in treatment approaches likely stems from the evolution of medical knowledge over time coupled with the absence of specific, well-designed studies. This situation underscores the need for standardized treatment protocols for this condition, which could potentially be more prevalent than currently reported.

As anticipated from our criteria, all cases presented with acute arterial ischemia following cocaine use. Notably, two of the cases involved patients who experienced intermittent claudication prior to the onset of acute ischemia. In terms of diagnostic findings, arterial thrombosis emerged as the most prevalent condition, identified in five patients (62.5%). Severe vasospasm was documented in one patient (12.5%). Additionally, ischemia/occlusion was reported in two patients; however, these reports did not specify whether the ischemia was thrombotic or secondary to severe vasospasm.

The treatments employed in these cases varied, encompassing a range of approaches. These included surgical thrombectomy in four patients, heparin anticoagulation in seven patients, intra-arterial vasodilators in one patient, intravenous vasodilators in two patients, balloon angioplasty in one patient, nerve block in one patient, intra-arterial anesthetic in one patient, and urokinase thrombolysis in one patient. Notably, anticoagulation with heparin was a common initial treatment across all patients. However, this approach was discontinued in the case reported by Debien et al. following the documentation of cerebrovascular hemorrhagic disease in the patient [20].

Post-treatment outcomes and follow-up observations varied across the cases. The data indicate that most patients showed improvement following treatment. Specific interventional endovascular treatments, including intra-arterial catheter placement, balloon angioplasty, and catheter thrombolysis, were used in three different cases, each resulting in varied outcomes such as necrosis, amputation, and improvement. Surgical thrombectomy was noted to yield the best results among the treatments; however, the limited sample size precludes any definitive conclusions. Furthermore, due to the diversity in patient profiles and treatments, establishing a correlation between demographic variables and treatment outcomes was not feasible with the current data set.

Discussion

This scoping review analyzed the existing evidence on the treatment of acute lower limb ischemia associated with cocaine use, focusing particularly on endovascular treatment. The limited research available, consisting primarily of case reports, precludes definitive conclusions and demonstrates a significant knowledge gap. Given the rising incidence of cocaine abuse and its association with serious vascular complications, particularly in individuals using high intravenous doses [22], there is a pressing need for more comprehensive studies with robust methodologies.

Comparison with Existing Literature

Our findings, including a male predominance and an average age of presentation of around 38 years, align with those reported by Zhou et al. [11]. Cocaine's role as a precursor to thromboembolic diseases is well-established, affecting primarily the central nervous system, heart, and acute peripheral occlusive artery disease [23]. The mechanism involves inhibition of norepinephrine reuptake and endothelial alterations, which lead to vasoconstriction and thromboembolic events [4,6,24-26]. This understanding supports the initial use of heparin in all reviewed cases. The variety of treatments, ranging from vasodilators to surgical thrombectomies, reflects the evolution of medical practices and the need to adapt to the specific context of each case. However, due to the diverse outcomes and limitations of the studies, generalizations are not feasible.

Clinical and Research Implications

These results point to a population at risk for acute lower limb ischemia associated with cocaine use, emphasizing the necessity for evidence-based guidelines and comprehensive, controlled studies for targeted therapeutic strategies. The multifactorial nature of vascular disease in cocaine users, often involving multiple substance abuse, complicates the direct attribution of causality to cocaine alone [27]. Additionally, the potential for acute embolization from foreign material in street cocaine should be considered in future research.

Limitations

The primary limitations of this review are the small and specific dataset, reliance on case reports, and the absence of longitudinal or controlled studies, which restricts the generalizability of our findings.

Recommendations for Future Research

Future research should involve multicenter studies with larger sample sizes and standardized methodologies. Comparative research across different treatment modalities is also needed. Insights from existing research on cocaine's impact on coronary and central nervous system disorders could inform the development of treatment strategies for acute lower limb ischemia. 

## Conclusions

Acute lower limb ischemia associated with cocaine use, though underreported, warrants significant clinical attention. This scoping review serves as a foundation for future studies with improved methodologies, aiming for a deeper understanding of the condition and enhanced therapeutic approaches. 

## References

[1] Biondich AS, Joslin JD (2016). Coca: the history and medical significance of an ancient Andean tradition. Emerg Med Int.

[2] Brody SL, Slovis CM, Wrenn KD (1990). Cocaine-related medical problems: consecutive series of 233 patients. Am J Med.

[3] Mabrouk MY, Guellil A, Haitam S, Deflaoui T, Jabi R, Bouziane M (2024). Peritonitis on sigmoidal perforation in a cocaine user: a rare case report. Int J Surg Case Rep.

[4] Kwentoh I, Daniel S, Atiku ES (2023). Cocaine-induced kidney, liver, lung, and muscle injury (C-KLM), presenting with foot drop: a case report. Cureus.

[5] Collins CG, Seoighe D, Ireland A, Bouchier-Hayes D, McGrath F (2008). Cocaine-associated lower limb ischemia. Vascular.

[6] Sharma T, Kumar M, Rizkallah A, Cappelluti E, Padmanabhan P (2019). Cocaine-induced thrombosis: review of predisposing factors, potential mechanisms, and clinical consequences with a striking case report. Cureus.

[7] Noël B (2001). Buerger disease or arsenic intoxication?. Arch Intern Med.

[8] Goldfrank LR, Hoffman RS (1991). The cardiovascular effects of cocaine. Ann Emerg Med.

[9] Alexander-Savino CV, Mirowski GW, Culton DA (2024). Mucocutaneous manifestations of recreational drug use. Am J Clin Dermatol.

[10] Fischer B, Coghlan M (2007). Crack use in North American cities: the neglected 'epidemic'. Addiction.

[11] Zhou W, Lin PH, Bush RL, Nguyen L, Lumsden AB (2004). Acute arterial thrombosis associated with cocaine abuse. J Vasc Surg.

[12] Shah R, Patel A, Mousa O, Manocha D (2015). Crack lung: cocaine-induced lung injury. QJM.

[13] Patel KH, Thomas KC, Stacey SK (2023). Episodic cocaine use as a cause of venous thromboembolism and acute liver injury. Am J Case Rep.

[14] Arksey H, O’Malley L (2005). Scoping studies: towards a methodological framework. Int J Soc Res Methodol.

[15] Levac D, Colquhoun H, O'Brien KK (2010). Scoping studies: advancing the methodology. Implement Sci.

[16] Tricco AC, Lillie E, Zarin W (2018). PRISMA extension for scoping reviews (PRISMA-ScR): checklist and explanation. Ann Intern Med.

[17] Peters MD, Godfrey C, McInerney P, Munn Z, Tricco AC, Khalil H (2020). Scoping reviews. JBI Manual for Evidence Synthesis.

[18] Colquhoun HL, Levac D, O'Brien KK (2014). Scoping reviews: time for clarity in definition, methods, and reporting. J Clin Epidemiol.

[19] McMullin CM, Bayat I, Rytina E, See TC, Varty K, Coughlin PA (2015). Profound acute limb ischemia affecting all four limbs following cocaine inhalation. J Vasc Surg.

[20] Debien B, Clapson P, Lambert E, Lenoir B, Perez JP, Pats B (2006). Acute cardiovascular complications of cocaine. About two case reports [Article in French]. Ann Fr Anesth Reanim.

[21] Seinturier C, Pichot O, Blaise S, Imbert B, Carpentier P (2006). Subacute ischemia of a lower limb and peripheral arterial toxicity of cocaine in a patient with juvenile arteriopathy [Article in French]. J Med Vasc.

[22] Sharma P, Ramirez-Florez S (2009). Consumption of cannabis and cocaine: correct mix for arterial occlusions. BMJ Case Rep.

[23] Mazzone A, Giani L, Faggioli P, Pichini S, Pacifici R (2007). Cocaine-related peripheral vascular occlusive disease treated with iloprost in addition to anticoagulants and antibiotics. Clin Toxicol (Phila).

[24] Havranek EP, Nademanee K, Grayburn PA, Eichhorn EJ (1996). Endothelium-dependent vasorelaxation is impaired in cocaine arteriopathy. J Am Coll Cardiol.

[25] Togna G, Tempesta E, Togna AR, Dolci N, Cebo B, Caprino L (1985). Platelet responsiveness and biosynthesis of thromboxane and prostacyclin in response to in vitro cocaine treatment. Haemostasis.

[26] Heesch CM, Wilhelm CR, Ristich J, Adnane J, Bontempo FA, Wagner WR (2000). Cocaine activates platelets and increases the formation of circulating platelet containing microaggregates in humans. Heart.

[27] Kumar PD, Smith HR (2000). Cocaine-related vasculitis causing upper-limb peripheral vascular disease. Ann Intern Med.

